# Early Transcriptome Differences Between Pre-Infected and Naïve Kid Goats Infected With *Haemonchus contortus*

**DOI:** 10.3389/fvets.2022.873467

**Published:** 2022-07-08

**Authors:** Hadeer M. Aboshady, Yoann Félicité, Julien Hira, Claude Barbier, Jean-Christophe Bambou

**Affiliations:** ^1^INRAE, ASSET, Petit-Bourg, France; ^2^Department of Animal Production, Faculty of Agriculture, Cairo University, Cairo, Egypt; ^3^INRAE, Plateforme Tropicale d'Expérimentation sur l'Animal, Petit-Bourg, France

**Keywords:** gastrointestinal parasite, goats, mucosal immunity, transcriptome, *Haemonchus contortus*

## Abstract

In small ruminant production, gastrointestinal nematode (GIN) infection is one of the major causes of economic losses. The aim of this study was to compare the abomasal mucosa transcriptome of naïve and pre-infected goats at early time points after *Haemonchus contortus* infection, in order to identify different pathways and upstream regulators involved in the host immune response. Naïve and pre-infected Creole kids were orally infected with 10,000 *H. contortus* infective larvae (L3), and abomasal mucosa was sampled at 0, 4, and 6 days post-infection (dpi). At 6 dpi, all the animals were slaughtered to perform parasite burden counts. The mean number of L4 recovered in naïve kids was more than twice as high as that recovered in the pre-infected ones (5,860 and 2,474 respectively, *p* < 0.001). RNA-seq analysis showed a number of differentially expressed genes (DEGs) very low for both naïve and pre-infected animals when comparing day 0 vs. day 4 post-infection. A total of 2,237 and 3,206 DEGs were identified comparing 0 vs. 6 dpi in naïve and pre-infected animals, respectively. Interestingly, only 18 DEGs were found for the comparison of pre-infected vs. naïve animals at 6 dpi. Ingenuity pathway analysis (IPA) showed that several immune responses were activated in pre-infected compared with naïve animals at 0 and 4 dpi such as Th2 and Th1 pathways, natural killer cell, B cell receptor, IL-2, and IL-15 signaling. On the other hand, both naïve and pre-infected animals showed activation for those pathways comparing 6 vs. 0 dpi, with no difference between them. A similar pattern was recorded for upstream regulator genes which were related to immunity like TNF, IL-1β, IL-2, IL-5, TGFβ1, IFNγ, TCR, IL-18, IL-6, and IL-4. Our results showed that at 0 and 4 dpi the immune response was activated toward Th1 and Th2 pathways in pre-infected kids compared to the naïve ones, however, the same immune response was developed in naïve kids as earlier as 6 dpi. We conclude that repeated *H. contortus* infection in kid goats induced a concomitant early activation of a Th1 and Th2 immune response resulting in the regulation of worm establishment.

## Introduction

Gastrointestinal nematodes (GIN) remain one of the major threats in sheep and goat production worldwide, both in temperate and tropical conditions. The negative impacts of these infections are both, direct and indirect on animal health, welfare, and performance (i.e., losses in milk, meat, and wool), with notably the impact of anthelmintic residues on soil biodiversity (1, 2). Furthermore, anthelmintic resistance is now reported worldwide, thus underlying the need for the development of sustainable and eco-friendly control strategies (3, 4). Host variation in resistance to GIN has been shown to be due to underlying genetic diversity which provides a promising control strategy through genetic selection (5, 6). Understanding molecular mechanisms underlying host response to GIN is the main step for developing appropriate genetic selection programs.

Numerous sheep studies showed that a strong Th2 response against GIN infections, characterized by parasite-specific local and systemic antibody response, is a key element for the development of a protective immune response and resistance to infection (7, 8). The fewer studies that have investigated the goat immune response to GIN, showed marked differences with the response in sheep (9, 10). RNA-sequencing (RNA-seq) technique is a powerful high-throughput next-generation sequencing technology for transcriptome analysis to identify and quantify genes expressed for complex quantitative traits like a response to infection (11). Indeed, numerous studies have been undertaken in sheep to identify genes and pathways, associated with the host response to GIN, from infected tissues and lymph node transcriptome (12–15). Results from these studies confirmed the role of the immune response, in particular the Th2 response for the development of host resistance to GIN. Meanwhile, the mechanisms identified in goats in response to GIN infections suggest a role of Th1, Th2, and TGF-ß1 genes (16).

The results from sheep studies have also shown that the response of naïve animals to GIN infection compared with pre-infected animals is qualitatively and quantitatively different (17–19). Thus, it has been suggested that the host response developed over time to infections, and the main immune mechanism involved is acquired and not innate (15). In goats, the difference between naïve and pre-infected animals in response to GIN infection and the underlying mechanisms has been poorly investigated.

Recently, it has been shown that the response during the early time of infection is a key component of the protective host-immune response in sheep (15, 20) and goats (16). Thus, studies focused on this early time after infection and comparing naïve and pre-infected animals need to be conducted. The objective of the present study was to characterize the mucosal host response in naïve and pre-infected goats to *Haemonchus contortus* infection at very early times after infection using RNA-seq technology.

## Materials and Methods

### Ethics Approval and Consent to Participate

All measurements and observations on animals were carried out in accordance with the current law on animal experimentation and ethics and approved by the French Ministry of Agriculture (authorization number: HC-69-2014-1) after evaluation by the Animal Care and Use Committee of French West Indies and Guyana (Comité d'Ethique en Matière d'Expérimentation Animale des Antilles et de la Guyane, C2EA-69).

### Animals, Management, and Experimental Design

This experiment was conducted at the INRAE PTEA (Plateforme Tropicale d'Expérimentation sur l'Animal) experimental farm in Guadeloupe (16° 20' North latitude, 61° 30' West longitude). All the animals owned by the INRAE PTEA were reared in this experimental farm since 1980. For this experiment, a total of 8 unrelated male kids, from 8 distinct sire families, were reared indoors and fed with parasite-free hay to avoid GIN infection. At 4- and 6-months-old, kids of the pre-infected group (*n* = 4) were experimentally infected with a single dose of 10,000 *H. contortus* L3 with a 10 ml syringe containing 10 ml of a suspension of L3 at 1,000 L3/ml in tap water. The naïve group (*n* = 4 animals) received 10 ml of tap water. After 5 weeks of infection for each challenge, the kids were drenched with moxidectine (Cydectine^®^, Fort Dodge Veterinaria S.A., Tours, France, 300 μg/kg). To avoid any bias due to moxidectine administration, naïve kids were drenched at the same time. For each challenge, Faecal Egg counts (FEC) was measured at week 4 and 5 post-infection. Approximately 10 g of feces were collected in plastic tubes directly from the rectum of each animal and transported from the experimental facility to the laboratory in refrigerated vials. The samples were individually analyzed using a modified McMaster method for rapid determination and FEC was expressed as the number of eggs/g feces. The mean FEC for the first and the second challenge of the pre-infected kids, at 4 and 6-month-old were 3567 ± 834 and 2765 ± 843, respectively. The FEC remained at zero for the naïve kids. At 8 months old, a fistula was surgically implanted in the abomasum of each animal to allow abomasal mucosa sampling at 0, 3-, and 6-days post-infection (dpi). At 9 months old, all the animals (pre-infected and naïves) were experimentally infected with a single dose of 10,000 *H. contortus* L3 as described above. Mucosal biopsies were collected at 0, 3-, and 6-days post-infection, then at 6 dpi, all the animals were humanly euthanized with a captive bolt stunning gun against their head, followed by exsanguination, in accordance with the procedures authorized in animal experiments. The abomasum was recovered for parasite counts.

### Surgical Procedure

The custom-designed abomasal cannula consisted of a flexible plastic tube with a length of 7 cm and a diameter of 2 cm with a rounded base of 4 cm in diameter. This flexible plastic was chosen to limit the possibility of mechanical abrasion of the mucosal surface of the abomasum. The animals were fasted 16 h before cannula insertion surgery. The animals were premedicated with ketamine (2 mg/kg Intravenous (IV), Le Vet Pharma, Wilgenweg, Netherlands), xylazine (0.2 mg mg/kg Intramuscular (IM), Le Vet Pharma, Wilgenweg, Neitherlands), and oxytetracycline (20 mg/kg IM, Eurovet Animal Health, Handelsweg, Neitherlands). The animals were positioned in left lateral recumbency. Skin over the surgical site was shaved and prepped with povidone iodine (Vétédine, Laboratoire Vetoquinol S.A., Lure, France). A ventral midline incision was made to locate and externalize the abomasum. A 3 cm purse-string suture (Silk 2–0) was placed midway between the lesser and greater curvature and a stab incision was made in the center to insert the cannula. Then, the purse-string suture was tightened and tied off. To maintain the abomasum in an anatomically correct position, another stab incision was made in the abdominal wall at 10 cm from the laparotomy incision on the right paramedian area to enable the cannula to be passed freely through. An external flange was placed over the external part of the cannula and fixed with an adhesive fabric plaster strip. A sterile compress was inserted into the cannula as a stopper. After the surgical procedure, all the animals were housed individually with free access to fresh water and hay.

### Biopsy Sampling Procedure

Biopsy specimens were taken from the abomasal mucosa using a flexible endoscope (FG-24V, Pentax, France). The biopsies samples of 2 mm × 2 mm × 2 mm taken with the endoscopic forceps with window (model KW1815S) were quickly snap frozen into liquid nitrogen and stored at – 80°C until RNA extraction. The animals were restrained in a harness made with a surgical drape allowing animal legs to protrude and which exposed the cannula. No sedation was used since no signs of discomfort or pain were observed during or after the procedure. The sterile compress inserted into the cannula was removed and the abomasal contents were collected. The endoscope was introduced into the abomasal lumen and 3 biopsies per animal and per time points were taken from the abomasal folds of the fundic mucosa. At each time point the whole fundic mucosa was observed and no sign of mucosal injury due to the previous sampling was observed.

### RNA Extraction and Sequencing

Total RNA was extracted using the NucleoSpin^®^ RNA isolation kit (Macherey-Nagel, Hoerdt, France) following the manufacturer's instructions, except that DNase digestion was performed with twice the indicated amount of enzyme according to our adjustments within our laboratory. The total RNA concentration was measured with NanoDrop 2000 (ThermoScientific TM, France). The RNA integrity was verified using an Agilent Bioanalyzer 2100 (Agilent Technologies, France) with an RNA integrity number > 8.5. The extracted total RNA was stored at – 80°C until sequencing.

High-quality RNA from all samples was processed for the preparation of cDNA libraries using an Illumina TruSeq RNA sample prep kit for mRNA analysis following Illumina's protocols. After quality control and quantification, cDNA libraries were pooled in groups of 6 and sequenced on 5 lanes on the HiSeqTM 2000 (Illumina^®^ NEB, USA) to obtain approximately 50 million reads (100 bp paired-end) for each sample with insert sizes ranging from 200 to 400 base pairs.

### Bioinformatics and Data Analysis

FASTQC was used to check the quality control of raw reads in FASTQ format and the Q20, Q30, and GC contents of the clean data were calculated. Transcript quantifications were made using the Salmon software (version 0.9.1) (21). The index was built within Salmon using NCBI RefSeq reference transcript of the *Capra hircus* genome (assembly ARS1). The reads from each sample were mapped to the same index and quantified. Unix commands were used to obtain corresponding gene and transcript identifiers from the NCBI RefSeq annotation of the *Capra hircus* (ARS1). Using these identifiers, the tximport (version 1.8.0) package was used to import data into the R software (v3.5.1) and summarize the TPM estimates obtained from the Salmon tool of all samples at the gene level (22). This process produced a global count file on which the statistical analyses were performed. Genes with low expression levels were removed using a threshold of greater than or equal to 5 counts across samples. Read counts table were used to identify differentially expressed genes (DEGs) using the Bioconductor package DESeq2 within R (23).

Seven comparisons were performed; three comparing pre-infected vs. naïve at day 0, 4, and 6 dpi, two comparing day 4 and 6 vs. 0 dpi in naïve kids, and another two comparing day 4 and 6 vs. 0 dpi in pre-infected kids. Genes were filtered using a Benjamini and Hochberg false discovery rate (FDR) of <0.001 to account for multiple testing. Final DEGs were determined on the basis of their fold change values to be log2 ≥ 1.0 for upregulated genes and ≤ −1.0 for downregulated genes. DEGs in each comparison were analyzed for canonical pathways and regulator effects using Ingenuity pathway analysis (IPA) software (Ingenuity Systems, Redwood City, CA). DEGs list of each comparison was uploaded to IPA and compared with humans orthologs data set generated by IPA to calculate the matching percentage.

### Parasite Counts

At slaughter (6 dpi, after biopsies sampling), the abomasum of each kid was isolated with contents for parasite counts. The contents of the abomasum were collected individually and the abomasum was washed with warm distilled water and scraped with a microscope slide to recover all established larvae. The contents and the wash water were stored at 4°C until counting. The parasites were collected, counted, and sorted according to the method previously described (24).

## Results

**Figure 1:**
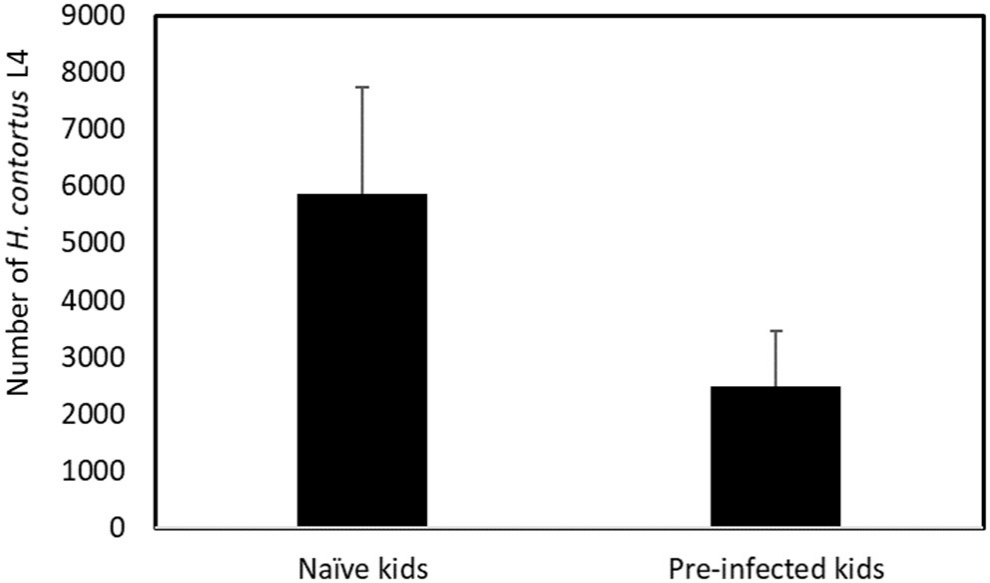
Mean number of *Haemonchus contortus* fourth–stage (L4) larvae recovered from naïve and pre–infected kids at 6 days post–infection. Means are significantly different (*p* < 0.001).

The number of L4 recovered from naïve and pre-infected kids at 6 dpi is presented in Figure 1. The mean number of L4 recovered in naïve kids was more than twice as high as that recovered in the pre-infected kid (5,860 and 2,474, respectively, *p* < 0.001). No other stage of larval development was found in either group.

RNA sequencing for each sample aligned to the reference *Capra hircus* genome (assembly ARSI) resulted in an average of 48.4 ± 1.8 million reads per sample. These reads correspond to 23,258 genes of the goat genome. A total of 17,311 out of the 23,258 annotated genes showed at least 5 read counts per raw and were used in the subsequent analysis.

Table 1 shows the number of DEGs for each comparison. Comparing pre-infected versus naïve kids, the number of DEG were 2,793 and 1,231 at 0 and 4 dpi, respectively. Meanwhile, at 6 dpi there were only 18 DEGs between pre-infected and naïve. In naïve kids, a low number of DEG was observed when comparing 4 and 0 dpi (12 DEG), in contrast, a high number of DEG was observed when comparing 6 and 0 dpi (2237 DEG). The same pattern was observed for pre-infected kids; a low number of DEG when comparing 4 and 0 dpi (9 DEG) and a high number of DEG when comparing 6 and 0 dpi (3206 DEG). Whatever the comparison, the number of upregulated and downregulated genes were similar, except for the comparison between pre-infected and naïve kids at 4 dpi, the number of downregulated genes was 218 compared with 1,013 for the upregulated ones. The range for log_2_-fold change was wider for the pre-infected kids comparing 6 and 0 dpi (-25.93 and 16.61) than the naïve kids for the same comparison (-9.60, 13.24). DEG from different comparisons showed human orthologs between 75 and 91%.

**Table 1:** The number of differentially expressed genes (DEGs) for the different comparisons including log_2_-fold change and the proportion of genes with human orthologs.

**Comparison**	**DEG**		**Log_**2**_-fold change**	**Human orthologs**
	**Up–regulated**	**Down–regulated**		
Pre–inf vs. Naïve 0 dpi	1618	1175	−12.03, 25.04	78.2%
Pre–inf vs. Naïve 4 dpi	1013	218	−7.40, 18.63	75.6%
Pre–inf vs. Naïve 6 dpi	8	10	−8.04, 2.81	77.8%
Naïve 4 vs. 0 dpi	11	1	−1.37, 6.99	91.6%
Naïve 6 vs. 0 dpi	1136	1101	−9.60, 13.24	83.4%
Pre–inf 4 vs. 0 dpi	9	0	3.18, 9.24	88.9%
Pre–inf 6 vs. 0 dpi	1331	1875	−25.93, 16.61	79.1%

*Pre–inf, Pre–infected kids*.

*dpi, days post–infection*.

Table 2 presents the gene symbol and log_2_-fold change for comparison showing a low number of DEG (pre-infected vs. naïve at 6 dpi, naïve 0 and 4 dpi, and pre-infected 0 and 4 dpi). No functional analysis was possible for these comparisons due to the low number of DEGs found. Nonetheless, the comparison of 4 and 0 dpi in naïve kids showed activation of the expression of different immune-related genes (e.g., TNFRSF12A, IL1B, CXCL8, and OLFM4; Table 2). Moreover, four genes involved in the IL-17 signaling pathways were found in this comparison: CXCL8, IL1B, MMP1, and MMP3. Meanwhile, pre-infected kids did not show the same pattern, and no pathways were suggested by the genes found. Besides, when comparing pre-infected and naïve kids at 6 dpi, most DEGs were not related to the immune response except MUC5AC, CCL20, KLRD1, and FAM3B.

**Table 2:** Differentially expressed genes (DEGs) and log_2_–fold change for pre–infected and naïve kids at 6 days post–infection (dpi), naïve and pre–infected comparing 0 and 4 dpi.

**Pre–inf vs. Naïve at 6 dpi**		**Naïve 4 vs. 0 dpi**		**Pre–inf 4 vs. 0 dpi**	
**Gene**	**Log_**2**_ -old change**	**Gene**	**Log_**2**_-fold change**	**Gene**	**Log_**2**_-fold change**
RPL9	−8.04	PARVB	−1.37	FER1L6	3.18
ACTB−1	−3.44	ITGA2	2.06	B3GNT6	4.65
MUC5AC	−3.37	TNFRSF12A	2.92	LOC106502720	4.70
CCL20	−3.05	PLAUR	3.00	VSIG1	4.80
LOC102180547	−2.64	IL1B	3.14	SLC6A14	5.48
RAB3B	−1.95	IFIT1	3.30	CHST4	5.49
FAM3B	−1.40	CDA	3.33	SOX21	7.78
TMC5	−1.36	MMP3	3.46	GKN1	8.83
DGAT2	−1.13	CXCL8	3.50	GKN2	9.24
VILL	−1.09	DIO2	3.62		
STMN2	1.15	MMP1	4.69		
IGF1	1.21	OLFM4	6.99		
MCTP1	1.29				
CPXM2	1.30				
TRPC4	1.36				
SLC2A3	1.63				
P2RX1	1.76				
KLRD1	2.81				

*Pre–inf, Pre–infected kids*.

*dpi, days post–infection*.

**Figure 2:**
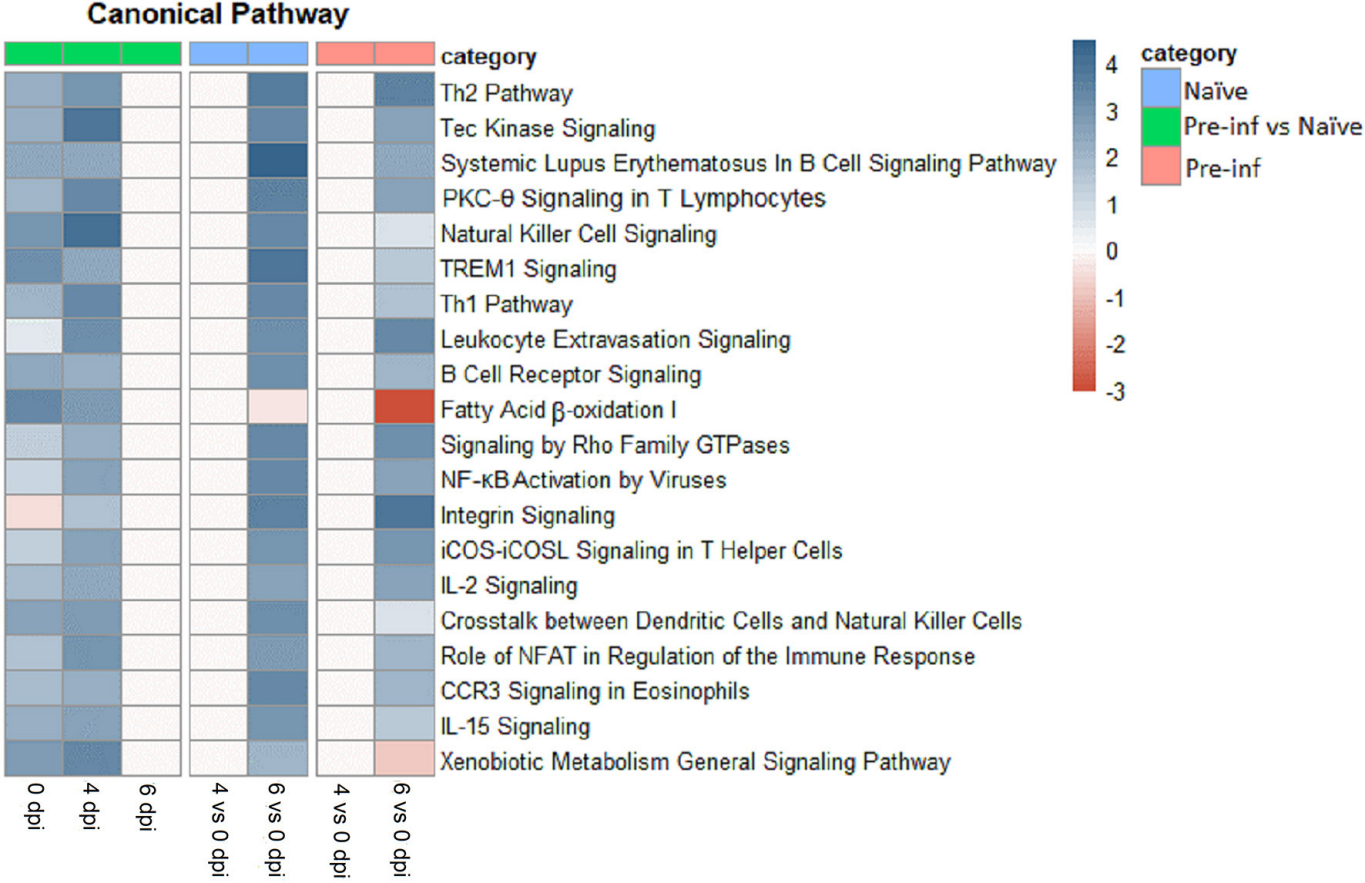
Top 20 canonical pathways of differently expressed genes for naïve and pre–infected (pre–inf) kids comparing 0 vs. 4 and 6 days post–infection and pre–infected vs. naïve at 0, 4, and 6 dpi.

The top 20 canonical pathways of DEG for different comparisons are presented in Figure 2. The majority of top canonical pathways were related to the immune response. Th1 and Th2 pathways were found in the top 10 pathways activated by 6 compared with 0 dpi, in both naïve and pre-infected kids. In general, all top canonical pathways had the same pattern in naïve and pre-infected kids when comparing different dpi. The activation of almost all pathways was higher in pre-infected compared with naïve kids at 0 and 4 dpi. However, no difference in pathways activated at 6 dpi was observed between pre-infected and naïve kids. The fatty acid β-oxidation pathway was highly downregulated in pre-infected animals when comparing 6 and 0 dpi, while it was upregulated comparing pre-infected and naïve at 0 and 4 dpi.

**Figure 3:**
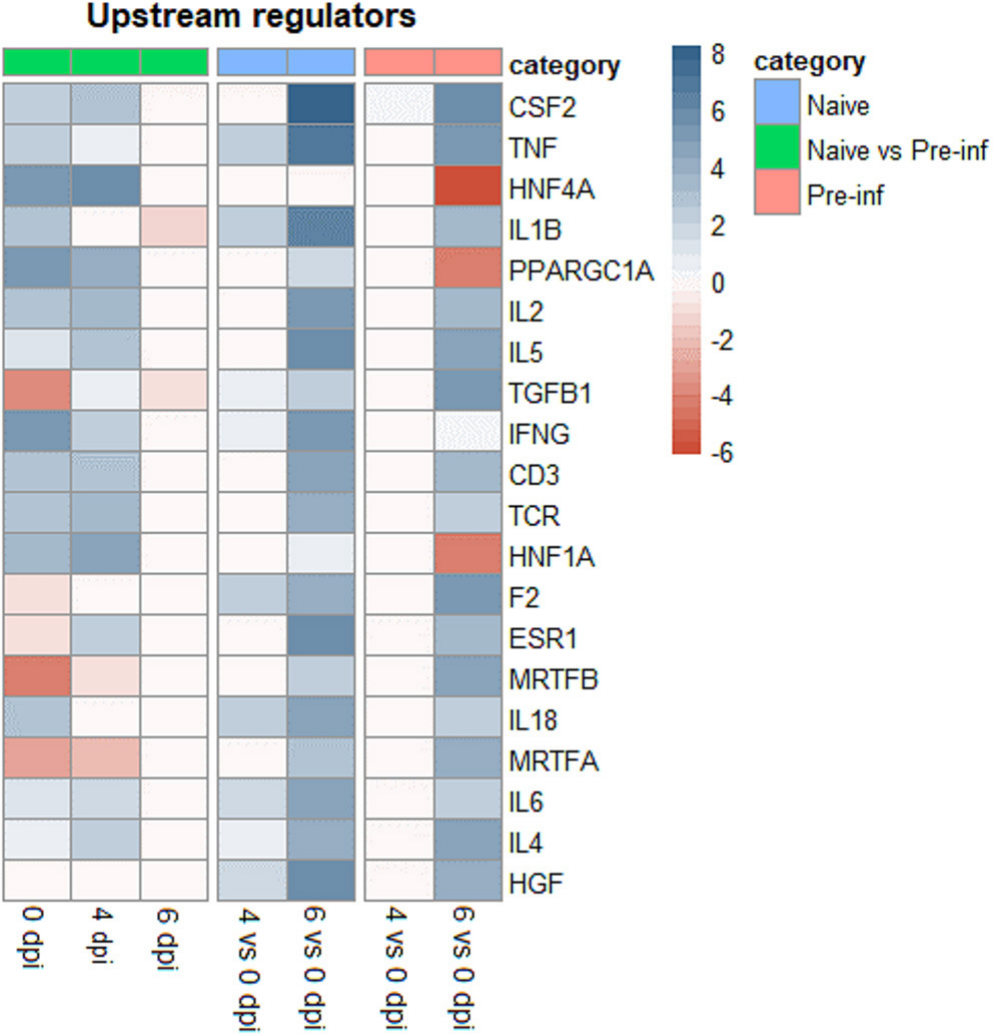
Top 20 upstream regulators of differently expressed genes for naïve and pre–infected (Pre–inf) kids comparing 0 vs. 4 and 6 days post–infection (dpi) and pre–infected vs. naïve at 0, 4, and 6 dpi.

The top 20 upstream regulators for different comparisons are presented in Figure 3. At 0 dpi pre-infected kids had already activated upstream regulators immune genes like TNF, IL1B, IL-2, IL-5, IFNG, CD3, TCR, IL-8, IL-6, and IL-4. On the other hand, these upstream regulators genes showed high activation in naïve animals when comparing 6 and 0 dpi, with activation score higher than those observed in pre-infected animals comparing the same days.

## Discussion

Numerous studies, mainly in sheep, suggested that the protective response against GIN infection would be a set of several parasite stage-specific mechanisms that differ both between breeds and divergently selected lines (resistant and susceptible) (25, 26). Thus, to better characterize the mechanism involved at the early stage of infection in goats, here were developed a model to monitor the same animals the response at 0, 3, and 6 dpi with *H. contortus*, and we compared naïve and pre-infected kids.

The lower establishment rate of infective larvae at 6 dpi observed in pre-infected kids suggested that a stage-specific mechanism developed after repeated infections targeted the early stage of infection. A similar study, comparing two lines of Merino sheep, divergently selected for resistance and susceptibility to GIN infections, was conducted recently in naïve and pre-infected lambs (27). Unfortunately, no measure of worm counts was performed, but the authors showed, that the number of DEG between the abomasal transcriptome of resistant and susceptible lambs was higher in the naïve compared with the pre-infected ones, on the third day after infection. In addition, the primary host response against *H. contortus* infection in the resistant line was characterized by the activation of several B cells-related canonical pathways. Indeed, many studies on sheep have shown that the major host-immune mechanism was acquired and not innate, with protective immunity developing over time in response to infection (15, 19, 28). In the present study, our results showed that the number of DEG between abomasal transcriptome of pre-infected and naïve kid goats was higher at the beginning of infection (day 0 and 4 post-infection), while there were very fewer DEGs between pre-infected and naïve goats at 6 dpi. These results are in accordance with previous findings showing a very low number of DEG at 3 dpi (ranging from 2 to 134), between primary and tertiary challenged sheep from two flocks selected for divergent responses to *H. contortus* and *Trichostrongylus colubriformis* (29). The slow development of the adaptive immune response or the high similarity of the first steps of the adaptive and primary immune responses have been hypothesized by the authors. Here, the first hypothesis would be unlikely given the lower establishment rate found in pre-infected kids. The high similarity of the adaptive and the primary immune response at 6 dpi is in fact observed, however, it is important to specify that it is at the transcriptional level. Further functional analysis (e.g., immunohistochemistry, proteomics) would have been necessary for a more detailed characterization of the mechanisms involved.

In pre-infected goats compared to the naïve ones, different immune pathways and upstream regulators were activated at the beginning of infection (0 and 4 dpi). Thereafter, at 6 dpi the same immune pathways and upstream regulators were activated in naïve goats. Our results comparing 6 and 0 dpi, showed that naïve and pre-infected animals a had high number of DEG involved in the activation of different immune responses including Th1, Th2, and B cell receptor signaling. The role of the Th1/Th2 balance in the development of a protective immune response against GIN infection has been previously discussed in sheep and goats (16, 30, 31). Sheep studies suggested that the Th1 response would be rather associated with susceptibility and the Th2 response with resistance (30, 31). Meanwhile, in goats, a concomitant activation of the Th1 and Th2 responses was observed both in resistant and susceptible animals, with only differences in the timing of the immune response between the two lines (16). Here, in line with this previous study, both Th1 and Th2 responses were activated in naïve and pre-infected animals, with earlier activation in pre-infected animals compared with the naïve ones. A similar pattern was recorded for upstream regulator genes which were related to immunity like TNF, IL1β, IL2, IL5, TGFβ1, IFNγ, TCR, IL18, IL6, and IL4.

Moreover, we recently identified genomic variants between resistant and susceptible goats to GIN, in 78% of genes controlling the T-cell receptor-signaling pathway (32). The T-cell receptor-signaling pathway would play a role in the development of host immunity against GIN infection. In this study, we found a B-cell receptor signaling pathway in the top activated pathway in naïve and pre-infected goats comparing 6 and 0 dpi. Thus, our results strongly suggested that in our biological model of Creole goats, receptor signaling pathways would be one key mechanism of the immune response against *H. contortus* activated both after the primary, or repeated infections. One difference between the immune responses after primary or repeated infection would be the kinetics of the mechanisms involved, with an earlier activation in pre-infected animals. Hence, we conclude that repeated *H. contortus* infection in kid goats induced a concomitant early activation of a Th1 and Th2 immune response resulting in the regulation of worm establishment.

## Data Availability Statement

The original contributions presented in the study are publicly available. This data can be found here: https://www.ncbi.nlm.nih.gov/biosample?LinkName=bioproject_biosample_all&from_uid=808243.

## Ethics Statement

The animal study was reviewed and approved by Comité d'Ethique en Matière d'Expérimentation Animale des Antilles et de la Guyane, C2EA-69.

## Author Contributions

HA and JCB conceived and designed the experiments, performed the bioinformatic and statistical analysis, and wrote the paper. JCB, YF, JH, and CB collected samples for the hematological and parasitological analysis. YF and JH performed the laboratory analysis. All authors read and approved the final manuscript.

## Funding

This study was funded by the Project MALIN (Maladies Infectieuses en Milieu Insulaire Tropical, La Région Guadeloupe and Fonds Européens FEDER). HA was supported by a doctoral fellowship from the Animal Genetics Division of INRAE and the European Project EGS-ABG (European Graduate School in Animal Breeding and Genetics).

## Conflict of Interest

The authors declare that the research was conducted in the absence of any commercial or financial relationships that could be construed as a potential conflict of interest.

## Publisher's Note

All claims expressed in this article are solely those of the authors and do not necessarily represent those of their affiliated organizations, or those of the publisher, the editors and the reviewers. Any product that may be evaluated in this article, or claim that may be made by its manufacturer, is not guaranteed or endorsed by the publisher.
